# 

**Published:** 1918

**Authors:** 


CONTENTS.

| | *Page* |
|---|---|
| **Some Developments in Abdominal Surgery.** By F. Lace, F.R.C.S. | 1 |
| **Editorial Notes** ... ... ... ... ... ... ... ... ... ... ... | 18 |
| **Obituary Notices :** | |
| John Michell Clarke ... ... ... ... ... ... ... | 23 |
| Harold F. Mole ... ... ... ... ... ... ... ... | 31 |

